# Selecting a multiplex PCR panel for accurate molecular diagnosis of intestinal protists: a comparative study of Allplex^®^ (Seegene^®^), G-DiaParaTrio (Diagenode^®^), and RIDA^®^GENE (R-Biopharm^®^) assays and microscopic examination

**DOI:** 10.1051/parasite/2022003

**Published:** 2022-02-09

**Authors:** Nicolas Argy, Céline Nourrisson, Ahmed Aboubacar, Philippe Poirier, Stéphane Valot, Adrien Laude, Guillaume Desoubeaux, Christelle Pomares, Marie Machouart, Yohann Le Govic, Frédéric Dalle, Françoise Botterel, Nathalie Bourgeois, Estelle Cateau, Marion Leterrier, Patrice Le Pape, Florent Morio, Sandrine Houze

**Affiliations:** 1 Parasitology-Mycology Laboratory, Bichat-Claude Bernard hospital, APHP 75018 Paris France; 2 IRD MERIT UMR 261, Pharmacy Faculty, Paris University 75006 Paris France; 3 Parasitology-Mycology Laboratory, CHU de Clermont-Ferrand, 3IHP, INSERM 63000 Clermont-Ferrand France; 4 Parasitology-Mycology Laboratory, CHU de Strasbourg 67000 Strasbourg France; 5 Parasitology-Mycology Laboratory, CHU de Dijon 21000 Dijon France; 6 Parasitology-Mycology Laboratory, Institut de Biologie, CHU de Nantes 44093 Nantes France; 7 Parasitology-Mycology-Tropical Diseases Department, CHU de Tours 37000 Tours France; 8 Parasitology-Mycology Laboratory, CHU de Nice 06000 Nice France; 9 Parasitology-Mycology Laboratory, CHU de Nancy 54511 Vandoeuvre-les-Nancy France; 10 Parasitology-Mycology Laboratory, CHU d’Angers 49100 Angers France; 11 Parasitology-Mycology Laboratory, CHU Henri Mondor, APHP 94000 Créteil France; 12 Parasitology-Mycology Laboratory, CHU de Montpellier 34295 Montpellier France; 13 Parasitology-Mycology Laboratory, CHU de Poitiers 86021 Poitiers France; 14 Microbiology Laboratory, CHD de la Roche-Sur-Yon 85000 la Roche-Sur-Yon France

**Keywords:** Multiplex PCR, Gastrointestinal protozoa, Molecular approach, PCR panel

## Abstract

Commercial multiplex PCR assay panels were developed to overcome the limitations of microscopic examination for parasitological diagnosis on stool samples. However, given the increased supply of this diagnostic approach, these assays must be evaluated to position them in a diagnostic algorithm. Analytical performances of the multiplex PCR assay G-DiaParaTrio, Allplex^®^ GI parasite and RIDA^®^GENE parasitic stool panel for detecting *Blastocystis* sp., *Entamoeba histolytica*, *Giardia duodenalis*, *Cryptosporidium* spp., *Dientamoeba fragilis,* and *Cyclospora cayetanensis*, were assessed through a retrospective comparative study on 184 stool samples initially sent for parasitological investigation. The composite reference method for parasitological diagnosis was microscopic observation and *Entamoeba histolytica*-specific adhesion detection when necessary. Multiplex PCR assays were performed on extracted DNA from each stool, following the manufacturer’s recommendations. Discrepant results with the composite reference method were investigated with species-specific PCR to approach a final parasitological diagnosis. Overall sensitivity/specificity for the multiplex PCR assays was 93.2%/100% for G-DiaParaTrio, 96.5%/98.3% for Allplex^®^ GI parasite and 89.6%/98.3% for RIDA^®^GENE, whereas the composite reference method presented an overall sensitivity/specificity of 59.6%/99.8%. These results confirmed the added diagnostic value of the multiplex PCR approach for gastrointestinal protists. Nevertheless, the PCR procedure and the analytical performance for each protist of interest, variable depending on the multiplex PCR assay, must be considered when implementing a PCR-based diagnostic approach.

## Abstract (fr)

Des panels commerciaux de tests PCR multiplex ont été développés pour dépasser les limites de l’examen microscopique pour l’examen parasitologique des selles. Cependant, compte tenu de l’offre croissante de cette approche diagnostique, ces tests doivent être évalués pour les positionner dans un algorithme de diagnostic. Les performances analytiques des tests PCR multiplex G-DiaParaTrio, Allplex^®^ GI parasite et RIDA^®^GENE parasitic stool panel pour la détection de *Blastocystis* sp., *Entamoeba histolytica*, *Giardia duodenalis*, *Cryptosporidium* spp., *Dientamoeba fragilis* et *Cyclospora cayetanensis*, ont été évaluées à travers une étude comparative rétrospective sur 184 échantillons de selles envoyés initialement pour un examen parasitologique. La méthode composite de référence pour le diagnostic parasitologique était l’observation microscopique et la détection d’adhérence spécifique d’*Entamoeba histolytica* lorsque cela était nécessaire. Des tests PCR multiplex ont été effectués sur l’ADN extrait de chaque selle conformément aux recommandations du fabricant. Les résultats discordants avec la méthode de référence composite ont été étudiés par PCR spécifique d’espèce pour approcher un diagnostic parasitologique final. La sensibilité/spécificité globale des tests PCR multiplex est respectivement de 93,2 %/100 % pour G-DiaParaTrio, 96,5 %/98,3 % pour Allplex^®^ GI et 89,6 %/98,3 % pour RIDA^®^ GENE alors que la méthode de référence composite présente une sensibilité/spécificité globale de 59,6 %/99,8 %. Ces résultats ont confirmé la valeur diagnostique ajoutée de l’approche PCR multiplex pour les protistes gastro-intestinaux. Néanmoins, la procédure de PCR et les performances analytiques pour chaque protiste d’intérêt, variables selon les tests PCR multiplex, doivent être prises en compte lors de la mise en œuvre d’une approche de diagnostic basée sur la PCR.

## Introduction

Gastrointestinal parasitic infections are a worldwide problem [8], characterized by a non-specific clinical presentation ranging from digestive discomfort to profuse diarrhea, depending on the pathogen involved. Countries with limited sanitation facilities are considered an area of high transmission of intestinal parasitic infection. Although probably underestimated, intestinal protists also cause significant illness in developed countries [9]. Whereas *Blastocystis* sp. and *Dientamoeba fragilis* are the most commonly detected, the most pathogenic intestinal protozoa are *Giardia duodenalis*, *Cryptosporidium* spp*.*, *Entamoeba histolytica* and *Cyclospora cayetanensis* [9, 13].

Morphological determination by light microscopic examination used to be the reference for parasitological diagnosis on stool samples. This approach presents limitations, since microscopy is a labor-intensive process with low sensitivity, and needs well-trained microscopists. In addition, *E. histolytica* specific diagnosis cannot be achieved by microscopy alone because of morphological similarities with the non-pathogenic species *E. dispar*/*E. moshkovskii/E. bangladeshi* [16].

Therefore, alternative diagnostic methods have been developed to overcome the limitations of conventional microscopic techniques. For *G. duodenalis* and *Cryptosporidium* spp*.* diagnosis, direct fluorescent antigen detection by trained microscopists has shown better analytical performances than conventional microscopy. Nevertheless, fluorescent microscopy is still time-consuming and requires skilled microscopists and appropriate equipment [16, 20]. Enzymatic immunoassays and immunochromatographic tests are also available, improving the identification of *E. histolytica* and the analytic turnaround time for *G. duodenalis* and *Cryptosporidium* spp*.* diagnostic. However, analytical performances of these tests vary according to the targeted parasites and manufacturers, with a high proportion of false-negative and false-positive results still requiring confirmatory tests [6, 7, 9, 16, 20, 28].

A deeper knowledge of inter-genera and species genetic variations associated with an increased possibility of molecular investigations are all reasons for the significant development of targeted PCR-based methods for diagnosing gastrointestinal parasitic diseases [32]. DNA-based detection methods rapidly found their place in the clinical parasitology diagnostic armamentarium. Importantly, these molecular approaches present increased sensitivity and specificity in low parasite prevalence populations, compared to microscopy approaches. DNA-based detection methods also allow multiplexing, which in turn facilitates the identification of co-infection in a high throughput screening condition [3, 11, 22, 25, 29, 31], in a staff and technical time cost-efficient fashion [26].

In recent years, the diagnostic market for gastrointestinal protists detection has grown significantly with an expansion of commercial multiplex diagnostic assays. These assays differ by several parameters, including the number of protists targeted and the detection technologies used [33]. In this context, specialized parasitology laboratories are responsible for evaluating commercial multiplex PCR in terms of analytical performances and practicability to better adapt these high-throughput multiplexed DNA detecting assays in a diagnostic algorithm for gastrointestinal protists [22, 26, 29].

In the present study, we assessed the performance of three commercial multiplex PCR kits, namely Allplex^®^ GI parasite assay (Seegene^®^), G-DiaParaTrio (Diagenode Diagnostics^®^) and RIDA^®^GENE parasitic stool panel (R-Biopharm^®^) for the detection of *G. duodenalis*, *Cryptosporidium* spp*.*, *E. histolytica*, *D. fragilis, C. cayetanensis* and *Blastocystis* sp. Compared with microscopy, the benefits of using these different targeted panels for optimal parasitological diagnosis are discussed in light of our results.

## Materials and methods

### Stool collection

A total of 184 stool samples were studied (Table 1), including 134 samples with mono or mixed infection with various protists, helminths or microsporidia, and 50 negative samples, all previously collected through a multicentric prospective study and managed as previously described [12]. Briefly, stool samples were prospectively collected during routine parasitological diagnostic procedures from patients suspected of gastrointestinal parasitic infection, at 12 French hospitals and shipped as soon as possible to Nantes University Hospital (principal investigating center) after the parasitological investigations. For each sample received, the initial parasitological diagnosis was confirmed by two trained microscopists using an iodine-stained wet mount and a Bailanger’s biphasic concentration method. In addition, detection of intestinal coccidia oocysts was performed using modified Ziehl–Neelsen staining. When *Cryptosporidium* spp*.* was detected, corresponding species were identified through the amplification and sequencing of the 18S rRNA [12]. Finally, for each stool sample positive for amoeba by microscopy in which *E. histolytica* identification was suspected, *E. histolytica*-specific adhesion was tested by ELISA (TechLab^®^, Blacksburg, VA, USA) for species identification. Upon reception, an aliquot from unpreserved stool was also performed and stored at −20 °C. Before DNA extraction, each stool sample was thawed and homogenized at room temperature. Approximately 200 mg of stool were resuspended in 1200 μL of liquid Amies medium (Copan Diagnostics Inc., Murieta, CA, USA) using nylon flocked swab and stored at −80 °C for 10 min. After thawing, for all included stool samples, DNA extraction was exclusively performed on a QIASymphony (QIAGEN, Courtaboeuf, France) using the complex 200 V6 DSP protocol with an 85-μL elution volume. DNA extracts were stored at −20 °C before this study.

**Table 1:** Pathogens included to evaluate the analytical performances of multiplex PCRs, identified by microscopy (iodine-stained wet mount, Bailanger’s method and modified Ziehl–Neelsen staining) completed by *E. histolytica*-specific adhesion for amoeba species only (composite reference method) [12].

Pathogen species identified by the reference method	Number of stool samples (*n* = 134)
*Blastocystis* sp.	17
*Chilomastix mesnili*	2
*Cryptosporidium hominis*	6
*Cryptosporidium parvum*	20
*Cryptosporidium felis*	1
*Cryptosporidium meleagridis*	1
*Cystoisospora belli*	2
*Dicrocoelium dendriticum*	1
*Dientamoeba fragilis*	1
*Endolimax nana*	21
*Entamoeba coli*	35
*Entamoeba dispar/moshkovskii*	12
*Entamoeba hartmanni*	4
*Entamoeba histolytica*	4
*Enterocytozoon bieneusi*	1
*Giardia intestinalis*	37
Hookworms	2
*Hymenolepis nana*	2
*Iodamoeba butschlii*	3
*Pentatrichomonas intestinalis*	1
*Sarcocystis* spp*.*	1
*Schistosoma mansoni*	4
*Taenia* spp*.*	1
*Trichuris trichiura*	1
Negative	50

### Real-time PCR detection

All three commercial multiplex PCR reagents (characteristics are given in Table 2) were used concomitantly in our laboratory, as recommended by the manufacturers. Allplex^®^ GI parasite assay (Seegene^®^, Seoul, South Korea) is a seven-plex PCR based on MuDT™ technologies. Results were interpreted using Seegene^®^ results processing software. G-DiaParaTrio (Diagenode Diagnostics^®^, Liège, Belgium) is a tri-plex real-time PCR performed on ABI 7500 (Thermofisher^®^, Waltham, MA, USA). RIDA^®^GENE parasitic stool panel (R-Biopharm^®^, Darmstadt, Germany) is a four-plex real-time PCR*.* On ABI 7500 (Thermofisher^®^), *Cryptosporidium* spp, *G. duodenalis* and *E. histolytica* were detected by hydrolysis probe, whereas *D. fragilis* amplified targets were evaluated by melting curve analysis. Inhibition of PCR amplification was assessed in each previous DNA extract by adding an internal positive control provided in each kit. DNA input was 5 μL for each multiplex PCR reagent. Each PCR experiment was validated according to the manufacturer’s recommendations. When PCR amplification was inhibited, the corresponding DNA extract was diluted at 1:10 and re-evaluated as described above.

**Table 2:** Characteristics of the three commercial multiplex PCR kits included in this retrospective comparative study for the assessment of gastrointestinal protists diagnosis.

Commercial kit	Manufacturer	Amplification technology	Thermocycler	Targets	Targeted genes	UDG system	Internal inhibition control#	Dedicated analysis software
Allplex^®^ GI parasite	Seegene	MuDT	Bio-Rad CFX96*	*Blastocystis* sp.	NA	Yes	Extraction and amplification control	Yes
				*Cryptosporidium* spp.				
				*Cyclospora cayetanensis*				
				*Dientamoeba fragilis*				
				*Entamoeba histolytica*				
				*Giardia intestinalis*				
G-DiaParaTrio	Diagenode	Taqman	Bio-Rad CFX96	*C. parvum*/*hominis*	Segment A	No	Extraction and amplification control	No
			Applied Biosystems ABI 7500*	*Entamoeba histolytica*	18S rRNA			
			Roche LC 480	*Giardia intestinalis*	18S rRNA			
			Qiagen Rotor-gene					
RIDAGENE parasitic stool panel	R-Biopharm	Taqman	Bio-Rad CFX96	*Cryptosporidium* spp.	ITS1-18S	No	Extraction and amplification control	No
		Melting curve	Applied Biosystems ABI 7500*	*Dientamoeba fragilis*				
			Roche LC 480	*Entamoeba histolytica*				
			Qiagen Rotorgene	*Giardia intestinalis*				
			Cepheid Smartcycler					
			Abbott m2000rt					
			Stratagene Mx3000P, Mx3005P					

MuDT Technologies is a real-time PCR technology that enables the detection of multiple targets with a Ct value in a single channel. NA: not available. UDG system: Uracil-DNA glycosylase system.

* Recommended thermocycler used in this study.

# Internal inhibition control was added into the reaction mixture before DNA amplification as recommended by the manufacturers to be used only as an amplification control in this study based on DNA extracts from a retrospective multicentric stool samples collection.

### Data analysis

Sensitivity and specificity were evaluated individually for each commercial multiplex PCR. Positive samples for parasites non-targeted by the commercial kits (amoeba species (*n* = 75), flagellates (*n* = 3), intestinal coccidian (*n* = 3), intestinal helminths (*n* = 11), and the microsporidia *Enterocytozoon bieneusi* (*n* = 1); Table 1) were used to determine each PCR assay specificity. Microscopic examination based on iodine-stained mount, Bailanger’s diphasic concentration method, modified Ziehl–Neelsen staining and *E. histolytica*-specific adhesion detection were considered the reference method (composite reference method). Multiplex PCR results were considered true positive or true negative when in agreement with the composite reference method. False-positive or negative results were defined as any discrepancy between the multiplex PCR and the composite reference method. In this case, discrepant results were secondarily investigated with specific commercial PCR targeted *E. histolytica* (R-Biopharm^®^) or specific in-house PCR assays for *D. fragilis* [21], *G. duodenalis* [30] and *Blastocystis* sp. [19]. The final parasitological diagnosis used to assess the sensitivity and the specificity of multiplex PCR was defined as the combination of composite reference method results or commercial multiplex PCR results with the specific confirmation PCR results.

## Results

PCR results were available for the 184 stool samples. PCR inhibition rates vary according to the PCR assays, from 0% for G-DiaParaTrio^®^, 2.2% (4/184) for Allplex^®^ to 8.2% (15/184) for RIDA^®^GENE. PCR inhibition was abolished for all these samples after 1:10 dilution of the extracted DNA. At least one targeted parasite was detected in 78/184 samples (42.4%), two protists were simultaneously detected in 34 (18.5%) samples, and three protists were detected in four stool samples (2.2%) according to the multiplex PCR panel. In all, 48 specimens were found to be negative with the multiplex assays.

Specifically, out of the 28 samples positive for *Cryptosporidium* by microscopy, 26 stool samples were also positive with the G-DiaParaTrio (6 *C. hominis* and 20 *C. parvum*), representing a sensitivity of 92.9% (Table 3). Two microscopy-positive samples were not detected, but these species were not included in the panel (1 *C. meleagridis* and 1 *C. felis*). Excluding these two stool samples, the sensitivity reached 100%. RIDA^®^GENE also detected 26/28 stool samples positive for *Cryptosporidium* (two false-negative results for *C. parvum*) leading to a sensitivity for *Cryptosporidium* spp*.* detection of 92.9% (26/28) and of 92.3% when considering only *C. parvum*/*C. hominis* targets (24/26) (Table 3). All 28 samples positive for *Cryptosporidium* were detected by Allplex^®^ GI parasite (i.e. sensitivity of 100%) (Table 3).

**Table 3:** Sensitivity and specificity estimated after a comparative study of commercial PCR multiplex for each targeted gastrointestinal protist.

Technique	No of samples tested	No of samples and corresponding sensitivity and specificity										Overall Se (CI)	Overall Sp (CI)
		*C. parvum/hominis*		*G. intestinalis*		*E. histolytica*		*D. fragilis*		*Blastocystis* sp..			
		Se (CI)*	Sp (CI)**	Se (CI)	Sp (CI)	Se (CI)	Sp (CI)	Se (CI)	Sp (CI)	Se (CI)	Sp (CI)		
G-DiaParaTrio^®^ (Diagenode)	184	100% (26/26)	100% (156/156)	92.5% (82.5–100) (37/40)	100% (144/144)	71.4% (42.9–100) (5/7)	100% (177/177)	NA	NA	NA	NA	93.2% (87.4–99) (68/73)	100% (477/477)
Allplex^®^ (Seegene)	184	100% (26/26)	100% (156/156)	97.5% (92.5–100) (39/40)	100% (144/144)	85.7% (57.1–100) (6/7)	100% (177/177)	100% (4/4)	94.4% (90.6–97.2) (170/180)	95.3% (89.1–100) (61/64)	97.5% (94.2–100) (117/120)	96.5% (93.4–99.5) (136/141)	98.3% (97.4–99.2) (764/777)
Rida^®^gene (r-biopharm)	184	92.3% (80.8–100) (24/26)	100% (156/156)	92.5% (82.5–100) (37/40)	99.3% (97.9–100) (143/144)	100% (7/7)	100% (177/177)	25% (0–75) (1/4)	98.3% (96.1–100) (170/180)	NA	NA	89.6% (82.8–96.4) (69/77)	98.3% (95.5–100) (646/657)
Composite reference method***	184	100% (26/26)	100% (156/156)	92.5% (82.5–100) (37/40)	100% (144/144)	42.9% (14.3–85.7) (3/7)	92.9% (79.4–100) (13/14)	25% (0–75) (1/4)	100% (180/180)	26.6% (15.6–37.5) (17/64)	100% (120/120)	59.6% (51.5–67.7) (84/141)	99.8% (99.5–100) (613/614)
Final parasitological diagnosis§		26		40		7		4		64		141	

Se: sensitivity, Sp: Specificity, CI: 95% confidence interval, NA: not applicable.

* Sensitivity corresponds to the percentage of true positive results obtained for the targeted pathogen by the tested technique and was calculated as follows: Se = (number of positive results observed/overall positive results). The 95% confidence interval was estimated with a binomial law.

** Specificity corresponds to the percentage of true negative results obtained by the tested technique and was calculated as follows: Sp = (number of negative results observed/overall negative results) */100. The 95% confidence interval was estimated with a binomial law.

*** The composite reference method was composed of a microscopic examination based on iodine-stained mount, Bailanger’s diphasic concentration method and modified Ziehl–Neelsen staining associated with *E. histolytica*-specific adhesion detection.

§ The final parasitological diagnosis corresponds to the combination of the composite reference method results or the PCR results obtained by a gastrointestinal multiplex PCR and the results obtained by the species-specific PCR targeted the same protists.

# Sensitivity and specificity for *C. cayetanensis* detection could not be evaluated because of the lack of positive samples.

For *G. duodenalis* detection, out of the 37 microscopy-positive samples, 34, 35 and 36 were detected by G-DiaParaTrio, RIDA^®^GENE and Allplex^®^, respectively. However, these PCR assays also yielded three, four, and three “false-positive” results, respectively of which three were further confirmed to be true positives by a *G. duodenalis* specific PCR and included as positive for the determination of the performance. Hence, compared with microscopy coupled to species-specific PCR, analytical performances (Se/Sp) for *G. duodenalis* detection were 92.5%/100%, 92.5%/99.3% and 97.5%/100% for G-DiaParaTrio^®^, RIDA^®^GENE and Allplex^®^, respectively. In comparison, microscopy for *G. duodenalis* presented sensitivity of 92.5% (Table 3).

Seventeen *E. histolytica*-specific adhesion detection assays were performed according to the diagnostic algorithm, among which four were positive. All three multiplex PCR assays detected correctly three *E. histolytica* microscopy-positive samples. The commercial *E. histolytica* species-specific PCR assay (R-Biopharm^®^) did not confirm the fourth positive *E. histolytica*-specific adhesion assay considered as *E. dispar*/*E. moshkovskii/E. bangladeshi.* Two, four and three additional positive results were observed for G-DiaParaTrio^®^, RIDA^®^GENE and Allplex^®^, respectively all confirmed as true positives by further investigations with the commercial *E. histolytica* species-specific PCR assay (R-Biopharm^®^). Lastly, analytical performances (Se/Sp) for *E. histolytica* detection were 71.4%/100%, 100%/100% and 85.7%/100%, for G-DiaParaTrio, RIDA^®^GENE and Allplex^®^, respectively (Table 3).

*Dientamoeba fragilis* observed by microscopy in a single stool sample was also detected by Allplex^®^ but not by the RIDA^®^GENE assay (not targeted by the G-DiaParaTrio assay). Strikingly, false-positive samples were obtained with RIDA^®^GENE and Allplex^®^ (4 and 13, respectively). In-house *D. fragilis* PCR confirmed 3 *D. fragilis* positive stool samples. Following these results, the adjusted sensitivity/specificity of RIDA^®^GENE was 25%/98.3% whereas Allplex^®^ detected all *D. fragilis* positive samples leading to 100% sensitivity with 94.4% of specificity (Table 3).

Among the 17 microscopy-positive *Blastocystis* sp. samples, 14 were also positive by the Allplex^®^ assay. However, this assay also detected *Blastocystis* sp. DNA in 50 additional stool samples. *Blastocystis* sp. specific in-house PCR assay was positive for 47 of them. Finally, adjusted analytic performances (Se/Sp) were 95.3%/97.5% for the Allplex^®^ assay (Table 3).

No positive stool sample for *C. cayetanensis* was observed by microscopy. In line with this, Allplex^®^ was also negative for all the 184 stool samples, representing specificity of 100%.

## Discussion

Protists are an important but underestimated cause of gastrointestinal infection in developed countries [9]. Despite several limitations, microscopic examination still represents the gold standard for diagnosing gastrointestinal parasites [16]. Sensitive multiplex PCR panels for molecular diagnosis of enteric protists have been developed to overcome microscopy-based diagnostic limitations [9, 13, 26]. Analytical performances of these new diagnostic tools need to be carefully assessed with two main objectives: the choice of the best-performing assay and the position of multiplex PCR in a diagnostic flowchart of intestinal parasitic infection [26, 29].

The present study evaluated three commercial multiplex PCR assay analytical performances compared to a composite reference method based on microscopic examination and *E. histolytica*-specific adhesion detection on a well-defined stool samples collection. According to our findings, these assays offer a higher detection rate of gastrointestinal protists regardless of the considered panel [1, 2, 10, 12, 14, 15, 18, 23], illustrated by an overall analytical sensitivity of 93.2% for G-DiaParaTrio, 96.5% for Allplex^®^ GI parasite and 89.6% for RIDA^®^GENE compared to 59.6% for microscopic investigation [25, 29]. These results should place molecular diagnosis as the first-line diagnosis for gastrointestinal parasites.

Interestingly, as observed in previous studies [1, 2, 12, 14, 15, 18, 23], analytical performances (Table 4) show high intra- and inter-panels variability for each targeted protist, probably explained by study design including the retrospective-prospective conception of the study, the type of the stool collection, the DNA extraction methods chosen, the PCR technologies used, the targeted genes, and the reference method chosen (PCR, ELISA, permanent stain) [14, 18, 20] (reviewed in Table 4). These observations are especially true for *E. histolytica*, *D. fragilis* [1] and *Blastocystis* sp., for which no clear diagnostic reference methods exist. Clearly, the possible low parasitic load, the rapid lysis of parasitic forms in the external environment, and the morphological polymorphism of parasitic forms observable may explain the poor performance of standard parasitological investigations. In addition, permanent staining is the reference method for *D. fragilis* detection, but this technique is not widespread in clinical parasitology laboratories. Moreover, to our knowledge, no complementary diagnostic tools (i.e., antigenic detection assays) were available for *D. fragilis* and *Blastocystis* sp. Contrary to *E. histolytica*, for which immunoassays exist but present poor performances [17]. All the reasons listed above could also explain how this comparative study revealed a higher detection rate of *E. histolytica*, *D. fragilis* and *Blastocystis* sp. by PCR [13, 16], highlighting the added value of molecular diagnosis as a first-line method to detect these parasites [2, 12, 18, 19, 23, 32]. However, the relevance of the molecular detection of these pathogens can be discussed. While *E. histolytica* is a well-known pathogenic protozoon that has to be treated [9], the pathogenic role of *D. fragilis* and *Blastocystis* sp. is still controversial [4, 13, 16, 24]. Whereas accumulated data seem to prove the pathogenicity of *D. fragilis*, positioning PCR on the front line for diagnosing dientamoebiasis [9, 13, 24, 30], the pathogenic role of *Blastocystis* sp. is still debated. Recently, *Blastocystis* sp. carriage has been correlated with dysbiosis in irritable bowel syndrome [4, 32].

**Table 4:** Review of previous comparative studies of commercial multiplex PCR panels targeting gastrointestinal protists.

Reference	PCR method	Study design	Samples (*n*)	DNA extraction	Amplification technologies	Gold standard	Protozoa Panel	Se (%)	Sp (%)
[23]	Easyscreen	Prospective	358	EZ1^®^ (Tissue kit and card)	Taqman on Cepheid^®^ smart cycler	Microscopy + PCR	*Blastocystis* sp.	96	100
							*Cryptosporidium* spp.	100	100
							*D. fragilis*	95	100
							*Entamoeba* spp.	92	100
							*G. intestinalis*	92	100
[12]	G DiaParaTrio	Retrospective	185	QIAsymphony^®^	Taqman on RotorGene	Microscopy + PCR	*C. hominis/C. parvum*	96	100
							*E. histolytica*	100	100
							*G. intestinalis*	92	100
[1]	G DiaParaTrio	Prospective	90	MagNA Pure 96	Taqman and melt curve on Light Cycler 480	Microscopy	*Cryptosporidium* spp.	55–100	100
	Ridagene						*G. intestinalis*	41–89	95–98
	BD max EPP			BD max	BD max		*D. fragilis*	71	97
[18]	G DiaParaTrio	Retrospective	126	QIAamp DNA stool mini kit	Taqman on RotorGene	PCR + Sanger sequencing	*Cryptosporidium* spp.	53.1–87.5	NA
	Ridagene			Speedtools DNA extraction	Taqman and melt curve on Mx3005P		*E. histolytica*	0–100	NA
				DNA Extract-VK					
	Allplex GI			Powerfecal DNA isolation	MuDT on CFX-96		*G. intestinalis*	68–100	NA
	FTD stool parasites			Wizard magnetic DNA purification					
[2]	Allplex GI	Retrospective	103	StarLab carry blair medium	MuDT on CFX-96	Microscopy	*Blastocystis* sp.	98.2–100	NA
							*Cryptosporidium* spp.	100	NA
							*C. cayetanensis*	100	NA
		Prospective	588				*D. fragilis*	80–81	NA
							*E. histolytica*	NA	NA
							*G. intestinalis*	81–100	NA
[14]	BD max EPP	Retrospective	391	BD max	BD max	Microscopy + PCR	*C. hominis/C. parvum*	95.5	99.6
		Prospective	2104				*E. histolytica*	100	100
							*G. intestinalis*	98.2	99.5

Abbreviations: FTD: fast track diagnosis, GI: gastrointestinal, EPP: enteric parasite pathogen, Se: sensitivity, Sp: specificity.

Conversely, because of efficient reference diagnostic techniques for their investigations, the diagnostic benefit of multiplex PCR for diagnosing other targeted digestive protist is more subject to discussion. It could be considered a complementary technique to the conventional methods used for digestive protist diagnosis. Among the genus *Cryptosporidium* spp*.*, *C. hominis* and *C. parvum* are the most frequently observed in humans and, by consequence, are the main targets of multiplex PCR panels. Inter-panel analytical sensitivities previously evaluated also present variations probably related to study design, PCR-based methods and type of species targeted (Table 4). DNA extraction is also a critical step for *Cryptosporidium* spp. detection whose yield varies among studies [27]. In this context of retrospective study including high-to-moderate *Cryptosporidium* spp. oocyst burden in stool samples, microscopy seems to present the same analytical sensitivity as multiplex PCR approaches, as already observed in previous studies [18]. These observations are mainly reported for *C. parvum*/*C. hominis* detection, but analytical sensitivities are more variable for other *Cryptosporidium* species which are not always targeted in the multiplex PCR panels. Other *Cryptosporidium* species represent approximately 5% of reported cases in Metropolitan France [5]. *Cryptosporidium* spp. molecular detection represented a real advantage, as observed in this study for *C. meleagridis* and *C. felis* targeted and correctly detected by RIDA^®^GENE and Allplex^®^. Early studies with simplex-specific PCR for *G. duodenalis* showed higher sensitivity compared to microscopic examination [26, 32], but the increased sensitivity was not observed with multiplex PCR assays (41–100%) [1, 2, 12, 14, 18, 23] (Table 4). In our comparative study, analytical performances for *G. duodenalis* detection are homogeneous between the panels tested with a sensitivity superior to 90%, which is quite similar to microscopic examination performances. Unfortunately, we could not evaluate the analytical performances for *C. cayetanensis* (no positive sample in our collection) as observed in a previous study [2]. *Cyclospora cayetanensis* involvement in food-borne poisoning epidemic clusters worldwide justified introducing this pathogen in multiplex PCR panels. Nevertheless, since *C. cayetanensis-*induced diarrhea has spontaneous resolution, this pathogen is poorly represented in samples collected in hospitalized patients.

Although the results presented here rely on a well-defined retrospective stool collection [12], the sample size is relatively weak, limiting the representativity of the analytical performances in the usual laboratory workflow. Therefore, prospective studies are necessary to confirm the findings presented here and allow for a broad field investigation and tested panel. Moreover, the stool sampling and DNA extraction methods used here were not recommended for all multiplex PCR panel kits, which could bias analytical performances by a non-optimal DNA yield. In fact, Seegene^®^ and R-Biopharm^®^ recommended NIMBUS^®^ technologies and RIDA^®^Xtract/Maxwell^®^ RSC, respectively for DNA extraction from stool samples. Our results must therefore be confirmed under conditions more faithful to those recommended. Finally, despite species-specific confirmation PCR in case of discrepancies, Sanger sequencing confirmation was not performed in parallel to avoid non-specific amplification results.

## Conclusions

Multiplex PCR approaches for gastrointestinal protist diagnosis have proven their superiority over microscopic examination because of higher detection rates and should be used as the first-line technique for gastrointestinal protists diagnosis [1, 16, 32]. However, combination with conventional diagnostic techniques could be discussed for Cryptosporidiosis because of a narrow *Cryptosporidium* species spectrum associated with few diagnostic advantages in comparison to microscopy. Comparative studies have revealed analytical performance variations according to multiplex PCR panels, proving the need to assess commercial gastrointestinal multiplex assay performances before implementation in clinical parasitology laboratories. The number and type of targeted protists, their corresponding analytical performances, DNA extraction methods compatibility, and the type of patient’ populations (adults/children, immunosuppression, community/hospital management) must be considered. Other variables such as the cost, workflow, and the need for additional equipment should be evaluated [20, 28]. However, at this time, no assessed multiplex PCR panel has presented 100% sensitivity for all protists and isolated positive results in asymptomatic patients have to be interpreted carefully [20]. Combining microscopy with a molecular approach is still debated due to the weak diagnostic gain in populations most exposed to gastrointestinal parasites in developed countries [29].

## Notes

**Cite this article as**: Argy N, Nourrisson C, Aboubacar A, Poirier P, Valot S, Laude A, Desoubeaux G, Pomares C, Machouart M, Le Govic Y, Dalle F, Botterel F, Bourgeois N, Cateau E, Leterrier M, Le Pape P, Morio F & Houze S. 2022. Selecting a multiplex PCR panel for accurate molecular diagnosis of intestinal protists: a comparative study of Allplex^®^ (Seegene^®^), G-DiaParaTrio (Diagenode^®^), and RIDA^®^GENE (R-Biopharm^®^) assays and microscopic examination. Parasite **29**, 5.
